# Investigating When, Which, and Why Users Stop Using a Digital Health Intervention to Promote an Active Lifestyle: Secondary Analysis With A Focus on Health Action Process Approach–Based Psychological Determinants

**DOI:** 10.2196/30583

**Published:** 2022-01-31

**Authors:** Helene Schroé, Geert Crombez, Ilse De Bourdeaudhuij, Delfien Van Dyck

**Affiliations:** 1 Department of Movement and Sports Sciences Faculty of Medicine and Health Ghent University Ghent Belgium; 2 Department of Experimental-Clinical and Health Psychology Faculty of Psychology and Educational Sciences Ghent University Ghent Belgium

**Keywords:** digital health, psychosocial determinants, health action process approach, physical activity, sedentary behavior, attrition, dropout, mobile health, healthy life style, health behaviors

## Abstract

**Background:**

Digital health interventions have gained momentum to change health behaviors such as physical activity (PA) and sedentary behavior (SB). Although these interventions show promising results in terms of behavior change, they still suffer from high attrition rates, resulting in a lower potential and accessibility. To reduce attrition rates in the future, there is a need to investigate the reasons why individuals stop using the interventions. Certain demographic variables have already been related to attrition; however, the role of psychological determinants of behavior change as predictors of attrition has not yet been fully explored.

**Objective:**

The aim of this study was to examine when, which, and why users stopped using a digital health intervention. In particular, we aimed to investigate whether psychological determinants of behavior change were predictors for attrition.

**Methods:**

The sample consisted of 473 healthy adults who participated in the intervention MyPlan 2.0 to promote PA or reduce SB. The intervention was developed using the health action process approach (HAPA) model, which describes psychological determinants that guide individuals in changing their behavior. If participants stopped with the intervention, a questionnaire with 8 question concerning attrition was sent by email. To analyze when users stopped using the intervention, descriptive statistics were used per part of the intervention (including pre- and posttest measurements and the 5 website sessions). To analyze which users stopped using the intervention, demographic variables, behavioral status, and HAPA-based psychological determinants at pretest measurement were investigated as potential predictors of attrition using logistic regression models. To analyze why users stopped using the intervention, descriptive statistics of scores to the attrition-related questionnaire were used.

**Results:**

The study demonstrated that 47.9% (227/473) of participants stopped using the intervention, and drop out occurred mainly in the beginning of the intervention. The results seem to indicate that gender and participant scores on the psychological determinants action planning, coping planning, and self-monitoring were predictors of first session, third session, or whole intervention completion. The most endorsed reasons to stop using the intervention were the time-consuming nature of questionnaires (55%), not having time (50%), dissatisfaction with the content of the intervention (41%), technical problems (39%), already meeting the guidelines for PA/SB (31%), and, to a lesser extent, the experience of medical/emotional problems (16%).

**Conclusions:**

This study provides some directions for future studies. To decrease attrition, it will be important to personalize interventions on different levels, questionnaires (either for research purposes or tailoring) should be kept to a minimum especially in the beginning of interventions by, for example, using objective monitoring devices, and technical aspects of digital health interventions should be thoroughly tested in advance.

**Trial Registration:**

ClinicalTrials.gov NCT03274271; https://clinicaltrials.gov/ct2/show/NCT03274271

**International Registered Report Identifier (IRRID):**

RR2-10.1186/s13063-019-3456-7

## Introduction

Digital health interventions have gained momentum to change health behaviors such as physical activity (PA) and sedentary behavior (SB) [1,2]. Their potential value lies in the ability to reach large groups in a personal, cost-effective, and time-efficient way [3-5]. Previous interventions have shown promising results in terms of behavior change [5-9]. Nevertheless, there are differences in use and completion [10-12]. It is important to ensure that participants do not drop out of the intervention. So far, attrition rates in digital health interventions are high (50% to 80%) [10,13,14]. As a result, interventions may lose part of their potential and accessibility. Also, effective evaluation of trials becomes a challenge. In order to reduce attrition, there is a need to investigate the reasons why individuals drop out. This question is often not addressed, as most studies focus on the effectiveness of interventions [15]. The answers may, however, provide valuable information in developing future digital health interventions [10]. In response to this problem, there has been a call for a science of attrition [10].

In order to understand attrition, 3 questions could be considered [10]. First, when do users stop using the intervention? Answers to this question may allow identification of weak parts of an intervention and may help in redesigning, restructuring, or removing certain parts. Most interventions only describe attrition rates at the end of the intervention. However, reporting attrition proportions at several time points can provide valuable information. For example, different patterns of attrition may occur: (1) a constant proportion of users may drop out of the intervention, (2) users may stay in the intervention first out of curiosity, which relates to the novelty effect (ie, the human tendency for heightened engagement to a novel phenomenon [16]), and then drop out when the novelty has worn off and eventually a stable group remains, (3) a group of users drops out of the intervention immediately and a stable group of users remains [10]. Each pattern could indicate different underlying causes of attrition.

Second, which users stop using the intervention? An answer to this question may direct researchers to tailor the content of the intervention to particular subgroups. Demographic variables such as being male [2,17,18], having a young age [17-21], having a lower educational level [22], and not having a partner [17] have been related to higher attrition rates in digital health interventions. The role of BMI in relation to attrition shows inconsistent results [21,23]. Also, the behavioral status of the participant at the start of the intervention may have an effect. Participants meeting the guidelines for moderate physical activity and for vegetable consumption at baseline showed lower attrition rates in comparison with those who did not meet these guidelines [23,24]. Davis and Addis [25] argued for investigation into the psychological determinants of behavior as predictors of attrition. Users with a low intention to change behavior have already been shown to drop out more often [26,27]. Accordingly, the role of other psychological determinants as predictors of attrition has not yet been fully explored.

Third, why do users stop using the intervention? Answers to this question may help researchers identify whether attrition is caused by features embedded in the intervention (eg, design of the intervention or technical problems with the intervention, lack of useful intervention content, too much questionnaires) or by reasons outside the intervention (eg, no interest in the topic, medical or emotional problems, lack of time).

In summary, the aim of this paper is threefold. The first aim is to examine when users stop using the intervention. The second aim is to investigate which users stop using the intervention informed by demographic variables, behavioral status at the beginning of the intervention, and psychological determinants. The third aim is to explore why users stop using the intervention by describing reasons for noncompletion.

This paper addresses these questions through secondary analysis of a digital health intervention that aimed to increase PA or reduce SB among the general population [28,29]. This intervention was developed using the health action process approach (HAPA) model, which describes psychological determinants that guide individuals in changing their behavior [30]. It is a 2-phase model that includes (1) motivational processes identified by determinants such as risk perception, outcome expectancies, and self-efficacy leading to a behavioral intention and (2) volitional processes identified by determinants such as action planning, coping planning, and self-monitoring bridging the gap between intention and the actual behavior [30]. As HAPA has been shown to effectively change behavior, the HAPA-based psychological determinants are considered important predictors of behavior change [30,31]. These predictors might not only influence behavior change but also the decision of whether to stop using an intervention.

## Methods

### Data Source

The data reported in this paper were from the MyPlan 2.0 factorial randomized controlled trial registered at ClinicalTrials.gov [NCT03274271] and approved by the Ghent University Hospital Ethics Committee. The protocol of the trial can be found elsewhere [28].

### Intervention

The MyPlan 2.0 digital health intervention consisted of a website and an optional mobile app to promote PA or reduce SB in healthy adults from the general population. MyPlan 2.0 was based on the HAPA model and consisted of a number of behavior change techniques (BCTs) aiming to influence participants’ HAPA-based psychological determinants of behavior change. The BCTs used in this study were goal setting, providing information on consequences of behavior, providing feedback on performance, social support, action planning, coping planning, self-monitoring, and reviewing behavior goals. These BCTs are described below.

Before the start of the intervention, participants chose which behavior (PA or SB) they wanted to improve (ie, goal setting). Depending on their choice, they were directed to the version of MyPlan 2.0 targeting PA or SB. The structure of the intervention was identical for the two behaviors.

**Figure 1:**
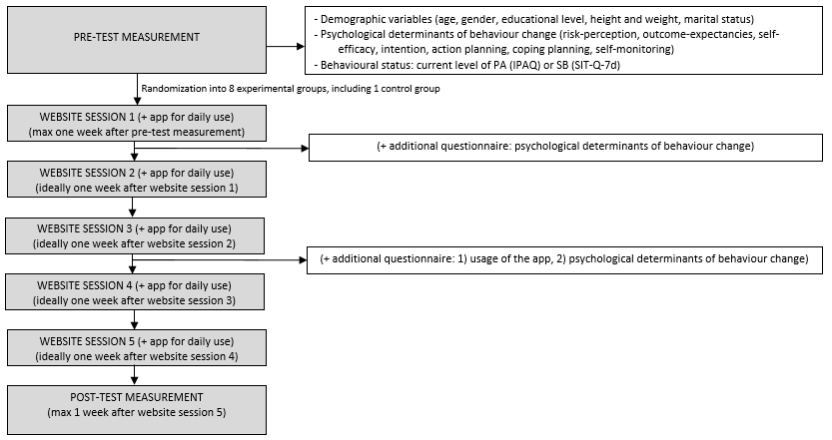
Flowchart of the MyPlan 2.0 intervention. IPAQ: International Physical Activity Questionnaire; PA: physical activity; SB: sedentary behavior; SIT-Q-7d: Last 7-day Sedentary Behavior Questionnaire.

The website is considered the main part of the intervention and consisted of 5 website sessions, with 1 week between each session (see Figure 1). Participants were expected to go through each of these sessions. The structure of the website sessions was fixed. In the first session, participants created a profile, were offered an optional quiz with information about the benefits of the selected target behavior (ie, providing information on consequences of behavior), and received tailored feedback on the current state of their chosen behavior (ie, providing feedback on performance). Thereafter, participants created an action plan by specifying how they wanted to reach their PA or SB goal, what they wanted do to, and where and when they wanted to do it (ie, action planning). Consequently, they identified potential barriers and thought about possible solutions (ie, coping planning). Thereafter, participants were prompted to monitor their behavior via the app or other options such as writing in their diary or on their calendar (ie, self-monitoring). At the end of the first session, they could read about how they could obtain social support from their partner, friends, family, or colleagues. In the 4 follow-up sessions, participants were asked to reflect on their progress of behavior change of the past week by evaluating their PA or SB goal (ie, reviewing behavior goals). They were also prompted to adapt or maintain their action plan, coping plan, and self-monitoring method. Screenshots of the website can be found in Multimedia Appendix 1.

The app was offered to participants as an optional tool to provide support on a daily basis. The app was synchronized with the website and developed as an extension to support users with their plans created in the website sessions. Use of the app was not mandatory. It consisted of 5 modules through which participants could freely navigate. In the first module, participants could again obtain a quiz regarding the benefits of more PA or less SB. In the second module, participants could review their action plan (which was created on the website) and change their plan throughout the week (ie, action planning). Moreover, the app reminded participants of their plan by sending notifications at scheduled times. In the third module, they could select barriers and receive an overview of possible solutions (ie, coping planning). In the fourth module, participants received a notification every evening to monitor their behavior by rating if they succeeded in their plan for the day on a scale from 0 to 5 (ie, self-monitoring). In the fifth module, users could collect medals by completing the website sessions, completing quizzes, and monitoring their behavior. These elements of gamification (ie, “the use of game design elements in nongaming contexts” [32]) were added to increase engagement with the intervention [33]. Screenshots of the app can be found in Multimedia Appendix 2.

### Intervention Content as Part of the Design

Intervention content differed as part of the design of the MyPlan 2.0 factorial randomized controlled trial [28]. Participants were randomly allocated to 8 different groups to evaluate the efficacy of 3 BCTs (ie, action planning, coping planning, and self-monitoring) and their combinations. As such, each group received a different version of the intervention, in which the 3 different BCTs were combined (Table 1). In both the website and the app, the BCTs could easily be removed or added in order to create the different groups [28]. Nevertheless, each participant received a basic intervention including the following BCTs: goal setting, providing information on consequences of behavior, providing feedback on performance, social support, and reviewing behavior goals [29].

**Table 1:** Different intervention content for each group as part of the design of the MyPlan 2.0 factorial randomized controlled trial.

	Action planning	Coping planning	Self-monitoring
Group 1	+^a^	+	+
Group 2	+	+	–^b^
Group 3	+	–	+
Group 4	–	+	+
Group 5	+	–	–
Group 6	–	+	–
Group 7	–	–	+
Group 8	–	–	–

^a^+: group received the intervention content including the behavior change technique.

^b^–: group received the intervention content without the behavior change technique.

### Participants and Procedure

The sample consisted of 473 participants who were recruited between February and December 2018 at the city library of Ghent or through social media. Inclusion criteria were a minimum age of 18 years, speaking Dutch, having internet access at home or work, and owning a smartphone (iOS or Android). Participants completed the 7 items of the Physical Activity Readiness Questionnaire as a screening instrument to detect individuals at risk for adverse effects when being more physically active [34]. Participants who answered no to all items were eligible for the study.

The flowchart for MyPlan 2.0 can be found in Figure 1. Participants completed pretest measurements including demographic variables, psychological determinants of behavior change, and questions assessing their current PA or SB level. When the pretest measurements were completed, participants were randomly allocated to 1 of the 8 different versions of the intervention (Table 1). Immediately after the randomization, participants could start with the intervention (maximum 1 week after the pretest measurements). The intervention consisted of 5 consecutive website sessions, ideally with 1 week between each session, and the optional mobile app, which could be used at any time during the intervention. Approximately 1 week after completing the last session, participants completed posttest measurements. The pretest measurements and posttest measurements were conducted via an online survey tool (Limesurvey GmbH).

Boosting strategies were used to encourage completion of each part (pretest and posttest measurements and the 5 website sessions): participants who did not complete a certain part after 1 week were sent a reminder, if they had not completed the part after 2 weeks, they were contacted by phone by the researcher (HS). If there was no response after 3 weeks, the participant was considered a noncompleter, and attrition was documented to have occurred during that specific part of the intervention. As such, the duration of the study could be different for each participant, depending on when they completed each part of the study.

After finishing website sessions 1 and 3, participants were invited to complete an additional questionnaire assessing psychological determinants during the intervention. After completing session 3, participants were also asked to complete another questionnaire assessing the use of the app [28]. Noncompletion of these additional questionnaires did not affect the continuation of the intervention. Data from these questionnaires were not used for analyses in this study.

### Measures

#### Definition of Attrition

In this paper, we differentiated between 2 types of attrition: (1) nonusage attrition, which refers to participants who were not using the intervention (ie, not completing the website sessions) and (2) dropout attrition, which refers to participants who were lost to follow-up because they stopped completing questionnaires for research purposes (ie, did not complete posttest measurements). Here, nonusage attrition automatically equaled dropout attrition because of the linear design of the study (eg, it was not possible to start, for example, with session 4 on the website if session 3 was not completed). Consequently, in this paper, we will just use the term attrition. Not using the app was not considered attrition because participants could use the app as an optional choice. Moreover, not completing the additional questionnaires after website sessions 1 and 3 was not considered attrition because it was still possible to proceed with the online website sessions.

#### Demographic Variables

At the pretest measurement, the following demographic variables were assessed: age, gender, education level (categorized as not having vs having a college/university degree), BMI (categorized as not overweight [≤25 kg/m²] vs overweight [>25 kg/m²]), and marital status (categorized as not having a partner vs having a partner).

#### Behavioral Status

The current level of PA or SB was assessed at pretest measurement to determine the behavioral status at the beginning of the intervention. For PA, the Dutch long version of the International Physical Activity Questionnaire [35] was used to measure moderate-to-vigorous intensity PA (MVPA) in minutes per week. For SB, the Dutch 7-day sedentary behavior self-report questionnaire [36] was used to measure total sedentary time in hours per day. Behavioral status at the beginning of the intervention was categorized as not meeting the guidelines (<150 minutes per week of MVPA or >8 hours per day of sitting time) versus meeting the guidelines (>150 minutes per week of MVPA or <8 hours per day of sitting time).

#### Psychological Determinants

The HAPA-based psychological determinants were measured at pretest measurement using a set of 26 items (ie, at least 3 items per determinant), which can be found in Multimedia Appendix 3. As described in our protocol paper [28], the set was based on the HAPA model and was iteratively developed and validated by an expert panel using cognitive interviewing [37,38] and a discriminant content validity method [39]. The same set of items was used for the 2 behaviors but the items were adapted to either PA or SB. Risk perception was assessed by 4 items; 1 of the items was “I am a person who is prone to high blood pressure.” The 4 items showed poor internal consistency (α=0.59). Removing the last item (“I am a person who is prone to have depression”) increased the internal consistency (α=0.71). Therefore, only the first 3 items were used to assess risk perception. Outcome expectancies were assessed with 5 items; 1 of the items for PA was “If I start being physically active regularly, I will feel better afterward.” Also here, the internal consistency was low (α=0.56). Removing the last item (“If I am physically active regularly, I have the feeling I lose time”) increased the internal consistency (α=0.68). As a result, only the first 4 items were taken into account to assess outcome expectancies. Self-efficacy was assessed by 5 items; 1 of the items for SB was “I am sure I can reduce my sitting time, even when I feel tired.” The items showed good internal consistency (α=0.83). Three items were used to assess intention; 1 of the items for PA was “I intend to be physically active regularly.” The internal consistency for these items was good (α=0.87). Action planning was assessed by 3 items; 1 of the items for PA was “I know exactly what to do (how, where, when, ...) to be physically active regularly.” All items showed good internal consistency (α=0.84). For coping planning, 3 items were used; 1 of the items for PA was “I already have thought about possible solutions in case I encounter obstacles in order to be physically active regularly (eg, if the swimming pool is closed, I go for a walk instead).” Also here, the items showed good internal consistency (α=0.88). Finally, 3 items were used to assess self-monitoring; 1 of the items for SB was “I am constantly monitoring how long I sit.” The internal consistency for these items was good (α=0.76). Participants rated all items on a 5-point response scale (1=totally disagree, 2=somewhat disagree, 3=neutral, 4=somewhat agree, 5=totally agree). For each determinant, the mean score of the items was used in the analyses.

#### Attrition-Related Questionnaire

When a participant was determined to be a noncompleter of a certain intervention part, a questionnaire with reasons for discontinuation was sent by email. Participants could indicate whether they found the reason for attrition totally not applicable, not applicable, neutral, applicable or totally applicable in response to 8 statements concerning attrition. The questions were based on attrition-related factors described in an article by Eysenbach [10].

### Statistical Analyses

#### Attrition Pattern

To analyze when users stopped using the intervention (aim 1), the numbers of participants per part of the intervention were described. For this paper, the different parts included the pretest and posttest measurements as well as the 5 website sessions (7 parts in total, see Figure 1). Each part was considered completed if participants completed the last question (for the pretest and posttest measurements) or visited the last page on the website (for the website sessions). Descriptive analyses were performed in Excel (Microsoft Corp).

#### Predictors of Attrition

To analyze which users stopped using the intervention, the following predictors of attrition were investigated: demographic variables, behavioral status, and psychological determinants at pretest measurement. Analyses were performed in SPSS (version 26, IBM Corp). Logistic regression models were fitted with attrition as a dependent variable at different time points (the number of the logistic regression models depended on the attrition pattern of aim 1). All independent variables (demographic variables, behavioral status, and psychological determinants) were entered separately into the logistic regression models. *P*<.05 was considered statistically significant, whereas *P* values between .05 and .10 were considered borderline significant; 95% confidence intervals were also reported.

In order to investigate whether different intervention content as part of the design of MyPlan 2.0 was a reason for attrition, 2 other predictors were added to the logistic regression models described above: the group to which the participants were allocated (group 1-8) and the choice of behavior participants wanted to improve (PA versus SB).

#### Reasons for Attrition

To analyze why users stopped using the intervention, the scores of participants to the attrition relation questionnaire were used. For each question, the number and percentage of participants who found the question (totally) not applicable, neutral, or (totally) applicable was shown. Descriptive analyses were performed in Excel (Microsoft Corp).

## Results

### Participant Characteristics

In total, 473 participants agreed to participate in the MyPlan 2.0 trial, completed pretest measurement, and were therefore considered users in this study. Of these participants, the mean age was 36.7 (SD 16.3) years, 69.1% (327/473) of participants were female, 66.4% (314/473) had a high level of education (college or university degree), 30.2% (143/473) were overweight, 42.3% (200/473) had a partner, and 49.0% (232/473) met the guidelines for either PA or SB. Descriptive statistics of the psychological determinants at pretest measurement are provided in Table 2. In addition, the number and percentage of participants in each group as well as the percentage of participants who chose PA or SB is provided (Table 2).

**Table 2:** Characteristics of participants of the factorial randomized controlled trial MyPlan 2.0.

Variable		Participants (n=473)
Age (years), mean (SD)		36.7 (16.3)
Gender (female), n (%)		327 (69.1)
Level of education (% high = university/college)		314 (66.4)
BMI (kg/m^2^), mean (SD)		23.5 (3.7)
Overweight, n (%)		143 (30.2)
Marital status (with partner), n (%)		200 (42.3)
Behavioral status (meets guidelines of PA^a^ or SB^b^), n (%)		232 (49.0)
Self-efficacy^c^, mean (SD)		3.54 (0.62)
Outcome expectancies^c^, mean (SD)		3.95 (0.49)
Risk perception^c^, mean (SD)		2.08 (0.66)
Intention^c^, mean (SD)		4.08 (0.55)
Action planning^c^, mean (SD)		2.84 (0.81)
Coping planning^c^, mean (SD)		2.47 (0.82)
Self-monitoring^c^, mean (SD)		2.06 (0.85)
**Participants in each group^d^, n (%)**		
	Group 1	59 (12.5)
	Group 2	60 (12.7)
	Group 3	56 (11.8)
	Group 4	56 (11.8)
	Group 5	61 (12.9)
	Group 6	59 (12.5)
	Group 7	61 (12.9)
	Group 8	61 (12.9)
Choice of behavior (participants who chose PA), n (%)		335 (70.8)

^a^PA: physical activity.

^b^SB: sedentary behavior.

^c^Mean on a score of 5 (SD).

^d^See Table 1 for information about each group.

### Attrition Pattern

**Figure 2:**
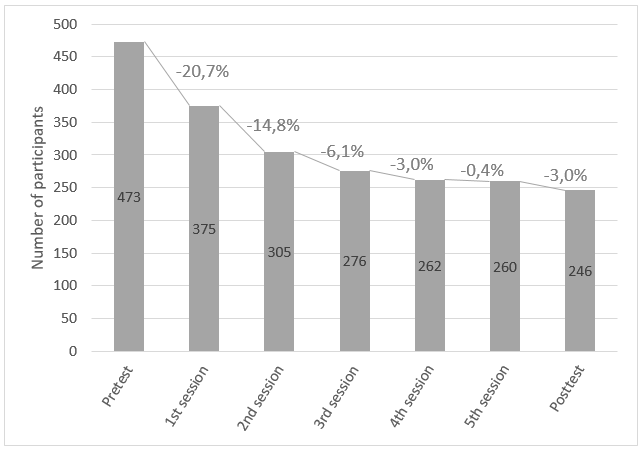
Attrition pattern of the intervention.

Of the participants, 47.9% (227/473) did not complete the intervention. Figure 2 shows the attrition pattern of the intervention. The biggest loss of participants was found in the early stage of the intervention; 20.7% (98/473) dropped out before completing the first website session, 14.8% (70/473) before completing the second session, and 6.1% (29/473) before the third session. This means that 41.6% (197/473) of participants dropped out before the third session and only 6.3% (30/473) after that session, which could determine a steady state of attrition after that part of the intervention.

### Predictors of Attrition

Predictors of attrition were investigated at 3 time points (Table 3). In order to do so, 3 logistic regression models were fitted: (1) identification of the predictors of first session completion, (2) identification of predictors of third session completion, and (3) identification of predictors of whole intervention completion (ie, completion of all 7 parts). This decision was based on the attrition pattern of aim 1: a large number of participants did not complete the first session, and a steady state of attrition was found after third session completion.

**Table 3:** Predictors of attrition.

Characteristics		First session completion (0=dropout before first session, 1=first session completion), OR^a^ (95% CI)	*P* value	Third session completion (0=dropout before third session, 1=third session completion), OR (95% CI)	*P* value	Whole intervention completion (0=dropout before posttest measurements, 1=whole intervention completion), OR (95% CI)	*P* value
**Demographic variables**							
	Age	1.0 (1.0-1.0)	0.91	1.0 (1.0-1.1)	0.21	1.0 (1.0-1.0)	0.16
	Gender (0=female, 1=male)	1.4 (0.8-2.3)	0.20	1.4 (1.0-2.2)	0.08	1.4 (1.0-2.1)	0.07
	Education (0=no college/university degree, 1=college/university degree)	1.2 (0.8-1.9)	0.46	1.2 (0.8-1.7)	0.46	1.0 (0.7-1.5)	0.89
	BMI (0=not overweight, 1=overweight)	0.9 (0.5-1.4)	0.57	0.8 (0.6-1.2)	0.38	0.9 (0.6-1.3)	0.52
	Marital status (0=no partner, 1=partner)	1.3 (0.8-2.1)	0.23	1.1 (0.8-1.6)	0.64	1.0 (0.7-1.5)	0.95
	Baseline norm (0=did not meet guidelines, 1=met guidelines)	1.1 (0.7-1.7)	0.81	1.0 (0.7-1.4)	0.80	1.0 (0.7-1.5)	0.95
**Psychological determinants**							
	Self-efficacy	1.0 (0.7-1.5)	0.86	1.2 (0.9-1.6)	0.30	1.1 (0.8-1.5)	0.54
	Outcome expectancies	1.1 (0.7-1.7)	0.62	1.1 (0.8-1.5)	0.63	1.0 (0.7-1.4)	0.84
	Risk perception	0.9 (0.7-1.2)	0.40	0.9 (0.7-1.2)	0.64	0.9 (0.7-1.2)	0.44
	Intention	1.0 (0.7-1.5)	0.98	1.0 (0.7-1.4)	0.98	1.0 (0.7-1.4)	0.84
	Action planning	0.8 (0.6-1.0)	0.08	0.8 (0.7-1.0)	0.09	0.9 (0.7-1.1)	0.25
	Coping planning	0.8 (0.6-1.0)	0.05	0.9 (0.8-1.2)	0.64	1.0 (0.8-1.2)	0.65
	Self-monitoring	0.8 (0.6-1.0)	0.19	0.8 (0.6-1.0)	0.04	0.8 (0.7-1.0)	0.08
**Intervention content as part of the design**							
	Group	1.0 (0.9-1.1)	0.45	1.1 (1.0-1.1)	0.22	1.1 (1.0-1.1)	0.18
	Behavior choice (0=participant chose PA^b^, 1=participant chose SB^c^)	1.2 (0.7-1.9)	0.52	1.3 (0.8-1.9)	0.26	1.2 (0.8-1.8)	0.29

^a^OR: odds ratio.

^b^PA: physical activity.

^c^SB: sedentary behavior.

#### Predictors of First Session Completion

No significant predictors of first session completion were found. However, the psychological determinants action planning and coping planning were found to be borderline significant (odds ratio [OR] 0.782 [95% CI 0.595-1.028], *P*=.08 and OR 0.769 [95% CI 0.590-1.002], *P*=.05, respectively), with participants with a higher score on action planning and coping planning being less likely to complete the first website session (Table 3).

Furthermore, the group to which participants were allocated and the choice of behavior participants wanted to improve as part of the design of MyPlan 2.0 were not significant predictors of first session completion.

#### Predictors of Third Session Completion

The psychological determinant self-monitoring significantly predicted whether participants completed the third session (OR 0.801 [95% CI 0.646-0.993], *P*=.04), with participants with a higher score on self-monitoring being less likely to complete the third website session. Furthermore, the demographic variable gender and the psychological determinant action planning were found to be borderline significant (OR 1.440 [95% CI 0.963-2.155], *P*=.08 and OR 0.822 [95% CI 0.655-1.032], *P*=.09, respectively). Men were more likely to complete the third website session, and participants with a higher score on action planning were less likely to complete the third website session (Table 3).

Furthermore, the group to which participants were allocated and the choice of behavior participants wanted to improve as part of the design of MyPlan 2.0 were not significant predictors of third session completion.

#### Predictors of Whole Intervention Completion

The demographic variable gender and the psychological determinant self-monitoring were found to be borderline significant (OR 1.437 [95% CI 0.969-2.13], *P*=.07 and OR 0.828 [95% CI 0.669-1.025], *P*=.08, respectively). Men were more likely to complete the whole intervention, and participants with a higher score on self-monitoring were less likely to complete the whole intervention (Table 3).

Furthermore, the group to which participants were allocated and the choice of behavior participants wanted to improve as part of the design of MyPlan 2.0 were not significant predictors of whole intervention completion.

### Reasons for Attrition

The reasons why participants stopped using the intervention were obtained from 51 of 227 participants (22% of all noncompleters) and can be found in Table 4. We were not able to contact the other participants. Participants who were older (OR 1.021 [95% CI 1.002-1.041]) and had a higher educational level (OR 2.938 [95% CI 1.345-6.418]) were more likely to complete the questionnaire.

The most endorsed reasons to stop using the intervention were “Filling out the questionnaires took a lot of my time” (28/51, 55%), “I don’t have time” (26/51, 50%), “The intervention doesn’t provide useful content” (21/51, 41%), “I experienced technical problems with the website or app” (20/51, 39%), “I already meet the health guidelines for PA/SB” (16/51, 31%), and “I experienced medical/emotional problems” (8/51, 16%).

The following reasons were reported not to be important to stop using the intervention: “I am not interested in the topic” (1/51, 2%) and “I don’t want to change my behavior” (1/51, 2%).

**Table 4:** Reasons for attrition (n=51).

I stopped using the interventions because...	(Totally) not applicable, n (%)	Neutral, n (%)	(Totally) applicable, n (%)
I am not interested in the topic	46 (90)	4 (8)	1 (2)
I don’t have time	19 (37)	6 (12)	26 (51)
I already meet the health guidelines for PA^a^/SB^b^	19 (37)	16 (31)	16 (32)
I don’t want to change my behavior	41 (80)	9 (18)	1 (2)
The intervention doesn’t provide useful content	25 (49)	5 (10)	21 (41)
Filling out the questionnaires took a lot of my time	16 (31)	7 (14)	28 (55)
I experience technical problems with the website or app	28 (55)	3 (6)	20 (39)
I experience medical/emotional problems	42 (82)	1 (2)	8 (16)

^a^PA: physical activity.

^b^SB: sedentary behavior.

## Discussion

### Principal Findings

This study investigated when, which, and why users stopped using an intervention to promote PA and reduce SB. The study demonstrated that 227 of 473 participants stopped using the intervention, and drop out occurred mainly in the first weeks. Certain predictors of first session, third session, or whole intervention completion were found. The most endorsed reasons to stop using the intervention were time-consuming nature of the questionnaires, not having time, dissatisfaction with the content of the intervention, technical problems, already meeting the guidelines for PA/SB, and, to a lesser extent, experiencing medical/emotional problems.

Although our intervention was systematically developed based on both qualitative and quantitative research [40,41] and boosting strategies were used to keep participants engaged with the intervention, an overall attrition rate of 47.9% was observed. This rate is similar to other interventions [10,13]. Attrition is an important obstacle in digital health interventions and should be reduced in order to increase the public health impact. The overall pattern of results indicates that the largest group of users drop out in the first weeks of the intervention, and a stable group of users remains after that. This pattern is frequently observed in digital health research [10,42] and is described as a L-shaped curve [10]. A possible explanation for this attrition pattern might be the time-consuming nature of the questionnaires, and participants reported this as a reason to quit the intervention. Indeed, at the start of the intervention, participants completed several questionnaires, mainly for research purposes and for tailoring advice throughout the intervention. Although we reduced the amount of questions substantially in comparison with a previous version of the intervention (MyPlan 1.0) to prevent attrition [18,43], participants still perceived it as too long. The review by Sharpe et al [44] showed that users are indeed less inclined to persevere with digital health interventions when they are found to be time-consuming and burdensome. Researchers should thus thoroughly reflect which and how many questions should be included in digital health intervention studies. Another option might be to collect baseline data through monitoring devices (eg, wearables such as Fitbit). Although this is often done for research purposes [45,46], such devices can also be used to provide tailored support in the beginning of an intervention (eg, tailored feedback on their current PA level).

According to Eysenbach [10], attrition might also be the result of a wrong user group, the members of which quickly lost interest. Indeed, the overall pattern of results indicates that participants already doing action planning, coping planning, and self-monitoring were more likely to drop out. As the MyPlan 2.0 intervention focused on these postintentional determinants [28], the intervention may not have added value for these participants as they might have been the wrong user group, causing them to stop using the intervention. However, one should be reminded that some of these effects were borderline significant in the current analyses, and thus await further replication and corroboration. Notwithstanding, an important question to answer is “Do participants already doing action planning, coping planning, and self-monitoring still benefit from an intervention?” On the one hand, one may reason that individuals who already have the competencies and skills to change behavior by themselves may not need additional support. On the other hand, it may well be that these individuals require a different, more individual approach that takes into account the needs and characteristics of the individual. Innovations in digital technology and artificial intelligence [47] enable researchers to develop more personalized interventions, making such an individual approach possible. Indeed, various studies indicate that personalization is crucial for future digital health interventions to increase engagement [44,48,49]. Yet, personalization can occur on different levels [48], and to the best of our knowledge, there is no consensus or framework on how to specifically personalize digital health interventions for PA or SB. Based on our findings, interventions may be personalized on 2 levels: dynamic tailoring of BCTs to the motivational stage to which an individual belongs and including personalized suggestions of BCTs at the operational level.

Regarding the first level, individuals may differ in terms of motivational stages: preintenders are individuals who do not yet have an intention to change, intenders are individuals who have an intention but do not yet act on these intentions, and actors are individuals who already act on their intentions [50]. Tailoring interventions to the stage of the participant may be more successful than mismatched interventions [51], but this tailoring often occurs only once at the beginning of an intervention. However, stages can also differ over time during the intervention (eg, intenders can become actors). By extension, research suggests [52,53] further differentiating between actors (individuals who recently started to perform the behavior) and maintainers (individuals who perform the behavior with high automatization over a long period of time). Intenders and actors may still benefit from BCTs such as action planning, coping planning, and self-monitoring, whereas maintainers might need other BCTs [29,52,54]. The findings of Schwarzer et al [55] indeed show that habitual activity does not require planning because the activity occurs rather automatically, whereas in the absence of the habit, planning appears to be a facilitator of PA. As such, providing dynamic tailoring of BCTs to these changing demands could reduce dropout in future interventions.

Determining the motivational stage of an individual is not an easy endeavor [56]. One might argue that individuals should be matched to stages based on meeting the health guidelines (eg, meeting the health guidelines may reflect being a maintainer and not meeting the health guidelines but having plans to work toward them may reflect being an intender). Accordingly, “Already meeting the guidelines for PA/SB” was one of the main reasons participants indicated stopping the intervention, with participants possibly needing other BCTs. However, meeting or not meeting the guidelines for PA/SB alone does not necessarily reflect the stage of the individual. One might also argue that individuals should be matched to stages based on the goal they have in mind. One can be a maintainer for a small behavior goal (eg, walking twice a week to work) and still be an intender or actor for a more challenging behavior goal (eg, running twice a week).

This brings us to the second level of personalization: including personalized suggestions of BCTs at the operational level. Participants who dropped out in our study might have been actors or maintainers who were looking for more challenging support. However, participants in our study were their own expert in terms of making action and coping plans, which means they had full control over the content of their plans. Although this is in line with self-regulation theory and increases autonomy [57], the delivery of these BCTs remained abstract and generic, offering standard but not personalized support. Indeed, it could be that participants were limiting themselves to plans that were already familiar to them, whereas they actually needed personalized suggestions that could provide them with new information and inspiration. Accordingly, dissatisfaction with intervention content was a reason for attrition in this study. This is in line with other research investigating user engagement [44]; participants in digital interventions for weight management most disliked generic information and repetition of content. As such, there is a need to tailor support at the operational level, involving suggestions of specific plans that are personalized to the individual. In addition, not having time was also found to be a reason for attrition. We acknowledge that thinking about action and coping plans is time intensive and requires high effort. Here, providing more personalized suggestions could result in a lower effort, time-effective intervention that could reduce dropout [58]. One should note, however, that behavior change in itself is not an easy endeavor, and raising awareness that behavior change takes time and effort is important. Here again, collecting objective data on PA/SB through monitoring devices [45] not only at the beginning but also throughout the course of the intervention will be important for personalization on both levels. That way, shifts between stages can be more easily identified (eg, intender to actor, actor to maintainer, actor to intender when there is a relapse). Passive data collection also offers the opportunity to provide more accurate personalized suggestions (eg, guiding participants from 8000 to 10,000 steps, guiding participants from walking to running, suggesting appropriate moments for a certain participant to do PA).

Remarkably, men were less likely to drop out than women. This is in contrast with previous findings [17,18,59]. A possible reason may be that this was an RCT compared to an open access study where participants had to give verbal consent for enrollment in the intervention. Several studies, including this one, have shown that men are less likely to enroll in studies compared to women [59], as women are more prone to respond in a socially desirable fashion [60]. However, once men do enroll in studies, they are more determined to complete the study. In order to increase engagement with the intervention, specific suggestions of plans as described in the previous paragraphs could also be personalized based on gender. Overall, most demographic variables did not predict whether certain subgroups of users stopped using the intervention. This could imply that the intervention can be broadly implemented and does not exclude specific target groups. Still, almost half of the participants dropped out of the study. This might indicate that other contextual and personal factors that were not investigated in this study (eg, social and physical environment, weather, location, mood, or health status) play a role.

Some other reasons not yet mentioned might explain why participants stopped using the intervention. First, although our website and app were thoroughly alpha and pilot tested [41], some technical problems were present at the beginning of the intervention that could have been a burden on participants. For example, our app did not work well with older smartphones, and the website caused technical problems when used in specific internet browsers (eg, Firefox did not always work well on tablets). Also, in the first weeks of the study, a bug caused the website to crash when a button for an optional website page was clicked on, preventing participants from returning to the main website page and completing their first website session. Future interventions should alpha test their websites and apps through all possible scenarios (various types of smartphones, internet browsers, laptops/tablets, etc) with a large user group. However, we should not assume that all technical problems can be solved in advance, as unforeseen barriers will always come up in digital health. Therefore, it may be useful if future interventions would provide a short manual with information to keep technical problems to a minimum (ie, press refresh when the website is stuck, use the internet browser Google Chrome, use the latest version of Android/iOS, do not use tablets). Second, some participants experienced medical/emotional problems during the intervention causing them to drop out. Future interventions should have the option to respond accordingly with particular advice or should refer to specific assistance (eg, doctor, psychologist, physiotherapist).

### Strengths and Limitations

This study has several strengths. To the best of our knowledge, this is the first study that investigated multiple psychological determinants of behavior as predictors of attrition in an intervention to promote an active lifestyle. Many studies have already described predictors of attrition in digital health but focused mainly on demographic variables [17,19,20,22] or factors relating to the digital intervention itself instead of the health behavior (eg, attitudes toward the digital tool, perceived control over the tool) [61]. Second, this study investigated attrition in different parts of the intervention, whereas most studies only describe attrition rates at the end of their interventions [10]. Third, this study had a large study sample.

This study also has a number of limitations. First, only a small proportion of users reported reasons why they stopped using the intervention (22% of all noncompleters). This low response rate could be explained by the format used to investigate reasons for dropout (eg, although it was stated in our protocol paper that telephone calls would be used, an online questionnaire was used due to lack of time). Future studies still may consider the use of telephone calls. In addition, most of these users dropped out before the third session of the intervention, making it impossible to compare reasons for attrition at the beginning of the intervention with those at the end of the intervention. Second, considering the linear design of the study (see Methods), no posttest measurements of noncompleters were collected. As such, it was not possible to explore whether noncompleters improved their PA or SB levels due to their (short) participation in the intervention. Investigating this could be important in future studies, as stopping the intervention does not necessarily coincide with failure [62]. Third, as this is an RCT, this study could have shown different results if it would have been an open access study (where attrition rates are usually even higher) [24,63]. Fourth, the study sample consisted mostly of women (69.1%) and highly educated adults (66.4%), which has also been the case in other digital health intervention studies [64]. As such, one should be careful when generalizing the study outcomes to a broader population.

### Conclusion

This study offered insights into when, which, and why users stop using a digital health intervention and provided some directions where future studies might focus on to prevent attrition. Personalization of interventions will be important, on one hand by dynamic tailoring of BCTs to the motivational stage to which an individual belongs and on the other hand by including personalized suggestions of BCTs at the operational level. Future studies should keep questionnaires (either for research purposes or tailoring) to a minimum by, for example, using objective monitoring devices, and technical aspects of digital health interventions should be thoroughly tested in advance.

## Appendix

Multimedia Appendix 1 Screenshots of the MyPlan 2.0 intervention website.

Multimedia Appendix 2 Screenshots of the MyPlan 2.0 mobile app.

Multimedia Appendix 3 A 26-item questionnaire on the Health Action Process Approach–based psychological determinants.

## Abbreviations

BCT: behavior change technique
HAPA: health action process approach
MVPA: moderate-to-vigorous physical activity
OR: odds ratio
PA: physical activity
SB: sedentary behavior
