# Supplementary material for: Investigating When, Which, and Why Users Stop Using a Digital Health Intervention to Promote an Active Lifestyle: Secondary Analysis With A Focus on Health Action Process Approach–Based Psychological Determinants
Source: JMIR Mhealth Uhealth. 2022 Jan 31;10(1):e30583. doi: 10.2196/30583 (PMC8845016; doi:10.2196/30583)
Supplement: Multimedia Appendix 2 [file mhealth_v10i1e30583_app2.pdf]

<u>Myplan 2.0. Screenshots of the mobile application</u>

Figure 1a. Module where participants could obtain quizzes regarding the benefits of more PA/less SB.

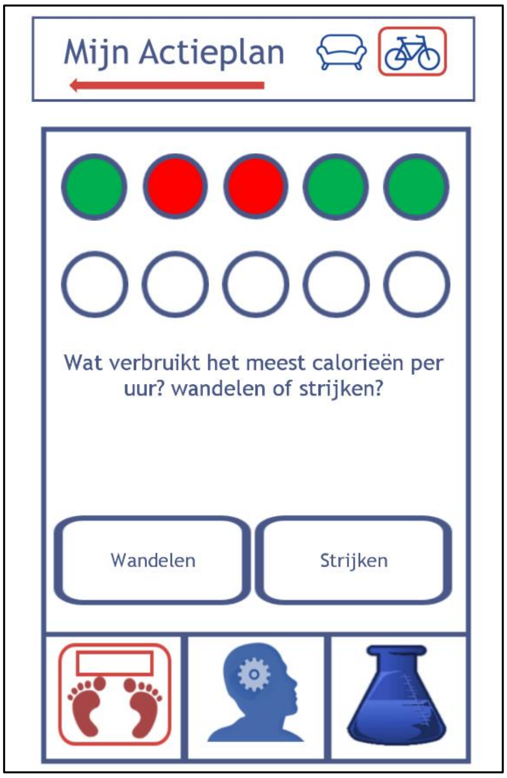

Translation:

"With which kind of movement you use the most calories? Walking or ironing?"

Figure 1b. Action planning module: revising and adapting plans to do more PA/less SB.

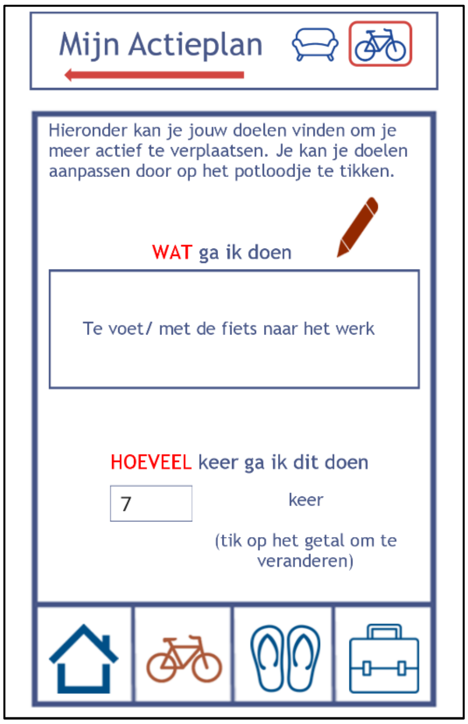

Translation:

"Below you can find your plans to do more active transport. You can adapt your plans by tapping on the pencil icon."

Figure 1c. Coping planning module: barriers and solutions for PA/SB.

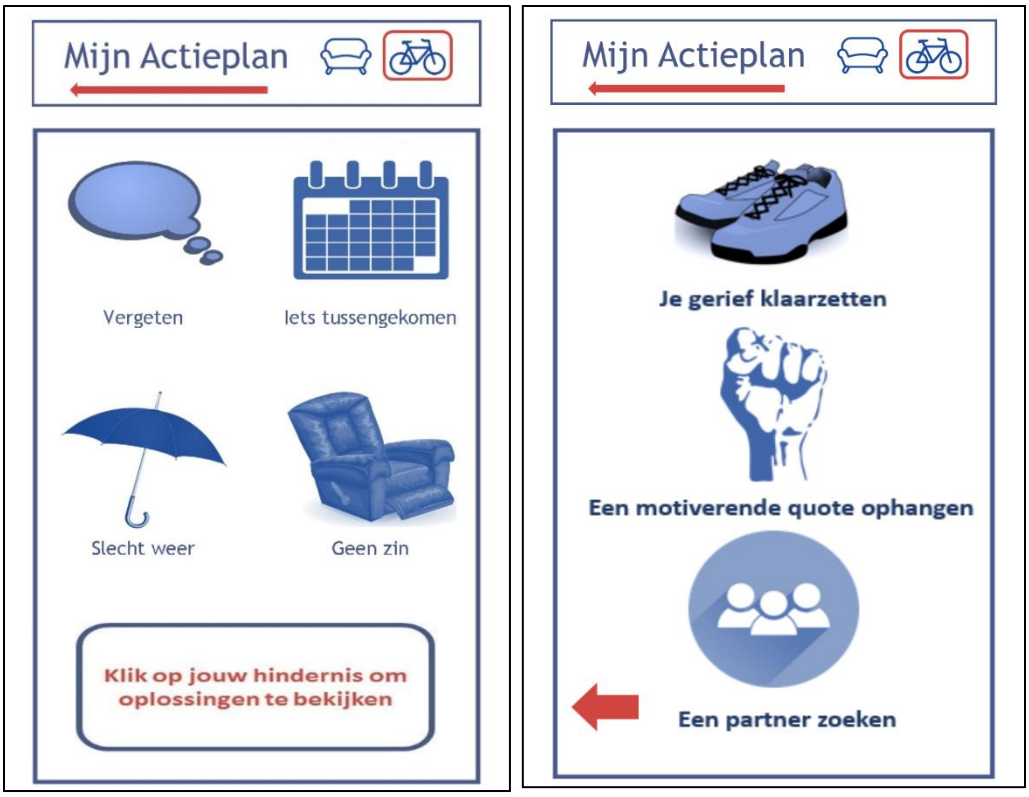

Text in red:
"Tap on a barrier to find solutions"

Figure 1d. Self- monitoring module: monitoring their PA/SB behaviour.

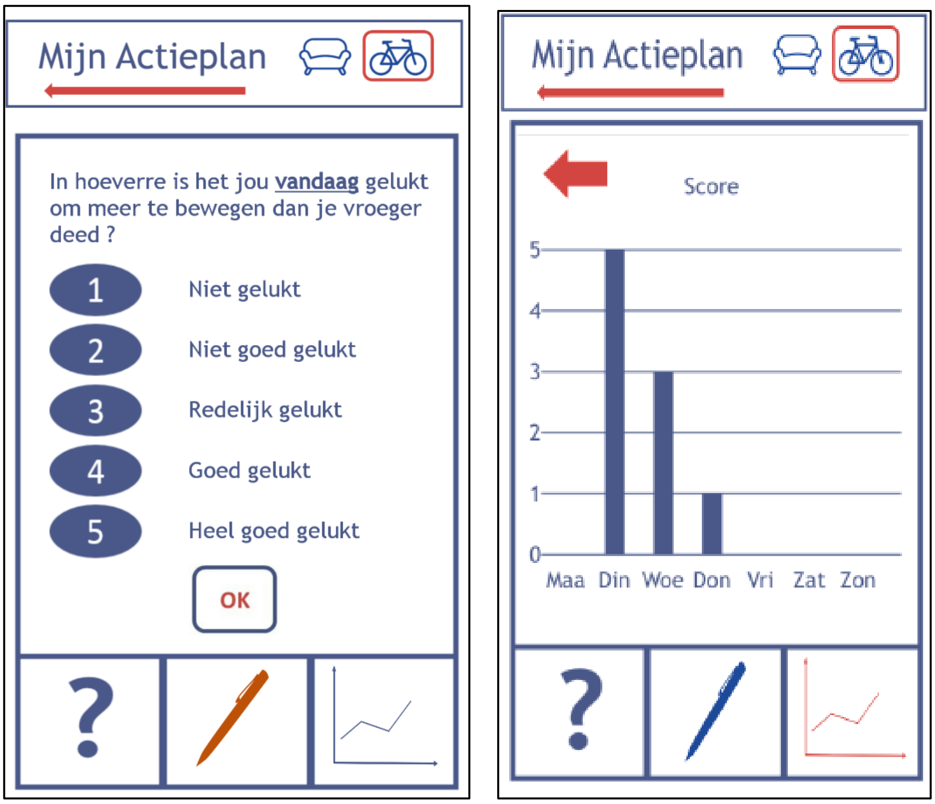

Translation:

"Today, to what extent did you move more than you did before?"

1= not succeeded

5= very well succeeded

Figure 1e. Module where participants could collect medals for completing website sessions, completing quizzes and monitor their behaviour.

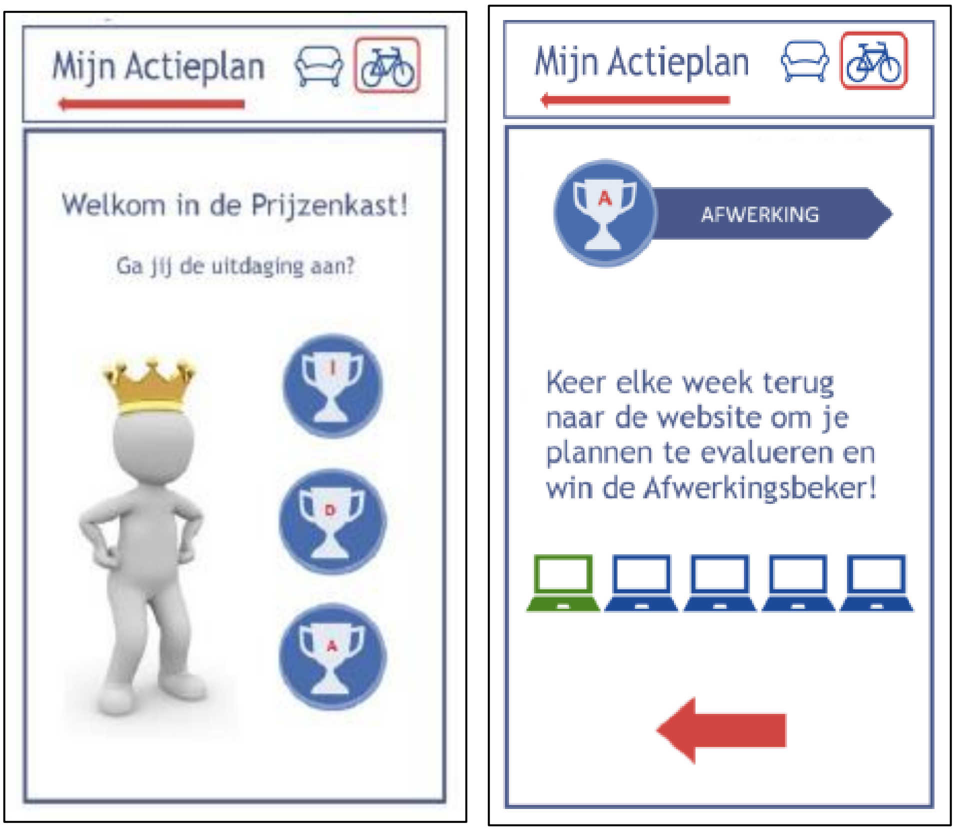

Left side: Overview of the medals participants could collect (left side).

Right side: Collecting points to win the medal for "finishing the website sessions".
