# Supplementary material for: Investigating When, Which, and Why Users Stop Using a Digital Health Intervention to Promote an Active Lifestyle: Secondary Analysis With A Focus on Health Action Process Approach–Based Psychological Determinants
Source: JMIR Mhealth Uhealth. 2022 Jan 31;10(1):e30583. doi: 10.2196/30583 (PMC8845016; doi:10.2196/30583)
Supplement: Multimedia Appendix 3 [file mhealth_v10i1e30583_app3.pdf]

Multimedia Appendix 3. Set of 26 items (in Dutch) for both physical activity and sedentary behavior of the HAPA-based psychological determinants.

Items Physical Activity	Items Sedentary Behavior	Answer	Reference
[Self-efficacy] Ik heb er vertrouwen in dat ik regelmatig kan bewegen	[Self-efficacy] Ik heb er vertrouwen in dat ik mijn zitten kan beperken	5 points respons scale<br><br>1= totally disagree,<br>2= somewhat disagree,<br>3= neutral,<br>4= somewhat agree,<br>5= totally agree	Schroé Helene, Van der Mispel Celien, De Bourdeaudhuij Ilse, Verloigne Maïté, Poppe Louise,<br>Crombez Geert. A factorial randomised controlled trial to identify efficacious self-regulation techniques in an e- and m-health intervention to target an active lifestyle: study protocol.Trials 2019, 20 (340).
[Self-efficacy] Ik heb er vertrouwen in dat ik regelmatig kan bewegen, ook als het soms moeilijk is	[Self-efficacy] Ik heb er vertrouwen in dat ik mijn zitten kan beperken, ook als het soms moeilijk is		
[Self-efficacy] Ik heb er vertrouwen in dat ik regelmatig kan bewegen, ook als dit heel wat tijd van me vraagt	[Self-efficacy] Ik heb er vertrouwen in dat ik mijn zitten kan beperken, ook als dit heel wat tijd van me vraagt		
[Self-efficacy] Ik heb er vertrouwen in dat ik regelmatig kan bewegen, ook als ik moe ben	[Self-efficacy] Ik heb er vertrouwen in dat ik mijn zitten kan beperken, ook als ik moe ben		
[Self-efficacy] Ik heb er vertrouwen in dat ik regelmatig kan bewegen, ook als ik dit soms enkele weken niet heb gedaan	[Self-efficacy] Ik heb er vertrouwen in dat ik mijn zitten kan beperken, ook als ik dit soms enkele weken niet heb gedaan		
[Outcome-expectancies] Ik zal me wellicht beter voelen als ik regelmatig beweeg	[Outcome-expectancies] Ik zal me wellicht beter voelen als ik mijn zitten beperk		
[Outcome-expectancies] Als ik regelmatig beweeg, zullen anderen me hiervoor waarderen	[Outcome-expectancies] Als ik mijn zitten beperk, zullen anderen me hiervoor waarderen		
[Outcome-expectancies] Regelmatig bewegen zal een positief effect hebben op mijn fysieke gezondheid (bv. bloeddruk, cholesterol, overgewicht, …)	[Outcome-expectancies] Mijn zitten beperken zal een positief effect hebben op mijn fysieke gezondheid (bv. bloeddruk, cholesterol, overgewicht, …)		
[Outcome-expectancies] Regelmatig bewegen zal een positief effect hebben op mijn mentale	[Outcome-expectancies] Mijn zitten beperken zal een positief effect hebben op mijn mentale		
[Outcome-expectancies] Als ik regelmatig beweeg, heb ik het gevoel dat ik tijd verlies	[Outcome-expectancies] Als ik mijn zitten beperk, heb ik het gevoel dat ik tijd verlies		
[Risk-perception] Ik ben een persoon die gevoelig is voor een hoge cholesterol	[Risk-perception] Ik ben een persoon die gevoelig is voor een hoge cholesterol		
[Risk-perception] Ik ben een persoon die gevoelig is voor een cardiovasculaire aandoening (bv. hartaanval, beroerte, …)	[Risk-perception] Ik ben een persoon die gevoelig is voor een cardiovasculaire aandoening (bv. hartaanval, beroerte, …)		
[Risk-perception] Ik ben een persoon die gevoelig is voor een hoge bloeddruk	[Risk-perception] Ik ben een persoon die gevoelig is voor een hoge bloeddruk		
[Risk-perception] Ik ben een persoon die gevoelig is voor een depressie	[Risk-perception] Ik ben een persoon die gevoelig is voor een depressie		
[Intention] Ik ben van plan om regelmatigte bewegen	[Intention] Ik ben van plan om mijn zitten te beperken		
[Intention] Ik heb me voorgenomen om regelmatig te bewegen	[Intention] Ik heb me voorgenomen om mijn zitten te beperken		
[Intention] Ik heb de intentie om regelmatig te bewegen	[Intention] Ik heb de intentie om mijn zitten te beperken		
[Action planning] Ik plan mijn beweging concreet op voorhand (bv. hoe, waar, wanneer...)	[Action planning] Ik plan het beperken van mijn zitten concreet op voorhand (bv. hoe, waar, wanneer...)		
[Action planning] Ik weet precies wat ik ga doen (bv. hoe, waar, wanneer...) om te bewegen	[Action planning] Ik weet precies wat ik ga doen (bv. hoe, waar, wanneer...) om mijn zitten te beperken		
[Action planning] Ik plan niet concreet op voorhand of ik ga bewegen	[Action planning] Ik plan niet concreet op voorhand of ik mijn zitten zal beperken		
[Coping planning] Ik heb al goed nagedacht over mogelijke oplossingen in het geval ik	[Coping planning] Ik heb al goed nagedacht over mogelijke oplossingen in het geval ik hindernissen		
[Coping planning] Ik heb al concrete oplossingen bedacht voor hindernissen die ik zou kunnen tegenkomen wanneer ik aan beweging wil doen	[Coping planning] Ik heb al concrete oplossingen bedacht voor hindernissen die ik zou kunnen tegenkomen wanneer ik mijn zitten wil beperken.		
[Coping planning] Ik weet precies wat ik moet doen als ik hindernissen ervaar om te bewegen	[Coping planning] Ik weet precies wat ik moet doen als ik hindernissen ervaar om mijn zitten te beperken.		
[Self-monitoring] Ik houd goed bij hoeveel ik beweeg	[Self-monitoring] Ik houd goed bij hoeveel ik zit		
[Self-monitoring] Ik noteer steeds op (bv. in mijn agenda, notitieboekje, in mijn gsm, …) hoeveel ik beweeg	[Self-monitoring] Ik schrijf steeds op (bv. in mijn agenda, in mijn gsm, …) hoeveel ik zit		
[Self-monitoring] Ik gebruik een stappenteller, app, activity tracker (bv. Fitbit) of iets anders om bij te houden hoeveel ik beweeg	[Self-monitoring] Ik gebruik een stappenteller, app, activity tracker of iets anders om bij te houden hoeveel ik zit		
